# Beyond Usual Geographical Scales of Analysis: Implications for Healthcare Management and Urban Planning

**DOI:** 10.1159/000527162

**Published:** 2023-01-10

**Authors:** Liliane Morais, António Lopes, Jorge Rocha, Paulo Jorge Nogueira

**Affiliations:** ^a^ISAMB − Instituto de Saúde Ambiental, Instituto de Medicina Preventiva, Faculdade de Medicina da Universidade de Lisboa, Faculdade de Medicina da Universidade de Lisboa, Lisbon, Portugal; ^b^Laboratório Associado TERRA, Faculdade de Medicina, Universidade de Lisboa, Lisbon, Portugal; ^c^Institute of Geography and Spatial Planning (IGOT), University of Lisbon, Lisbon, Portugal; ^d^Área Disciplinar Autónoma da Bioestatística (laboratório de Biomatemática), Instituto de Medicina Preventiva, Faculdade de Medicina da Universidade de Lisboa, Lisbon, Portugal; ^e^CIDNUR − Centro de Investigação, Inovação e Desenvolvimento em Enfermagem de Lisboa, Escola Superior de Enfermagem de Lisboa, Lisbon, Portugal; ^f^National School of Public Health (CISP), New University of Lisbon, Lisbon, Portugal; ^g^NOVA National School of Public Health, Public Health Research Centre, Universidade NOVA de Lisboa, Comprehensive Health Research Center (CHRC), Lisbon, Portugal

**Keywords:** Public health, Healthcare management, Policymakers awareness, Geographic scales, Cardiorespiratory mortality, Saúde pública, Gestão dos cuidados de saúde, Conscientização dos decisores, Escalas geográficas, Mortalidade cardiorrespiratória

## Abstract

**Introduction:**

In the context of climate emergency, advances in geographic information systems, geocoding, and geomedicine allow us to go beyond the conventional usual scales and be aligned with people's needs, improving knowledge and accuracy of the spatial pattern of health outcomes. This study shows that the geographical scale of analysis affects the interpretation of health outcomes.

**Methods:**

All mortality that occurred in Portugal in 2014–2017 was geocoded. From 435,291 addresses, 412,608 were geocoded with success. As an example, we use the spatial patterns of the elderly's heat-related cardiorespiratory mortality.

**Results:**

It is shown: (i) it is possible to have high quality and accuracy of spatial data used in health outcomes analysis; (ii) how geographic scales reveal different degrees of detail in health outcomes analysis; (iii) the neighbourhood scale revealed different patterns of cardiorespiratory mortality from the usually available scale (parish).

**Discussion:**

Our findings suggest the relevance of geocoding health outcomes with a finer scale in tackling the challenges of the healthcare sector, and in support of planning decision-making, closely matching citizens' needs. Without running the risk of losing potentially major prospects, better healthcare management is achievable, with optimal resource allocation, and improved detailed and informed policymaking, allowing enhanced climate health equity in cities promotion.

## Resumo

**Introdução:**

No contexto da emergência climática, os avanços nos Sistemas de Informação Geográfica, a geocodificação e a geomedicina, permitem ir além das escalas convencionais e alinharmo-nos às necessidades das pessoas, melhorando o conhecimento e a precisão do padrão espacial dos resultados de saúde (*health outcomes*). Este estudo mostra como a escala geográfica de análise afeta a interpretação dos resultados de saúde.

**Métodos:**

Toda a mortalidade ocorrida em Portugal em 2014–2017 foi geocodificada. De 435.291 endereços, 412.608 foram geocodificados com sucesso. Como exemplo, foram utilizados os padrões espaciais da mortalidade cardiorrespiratória associada ao calor nos idosos.

**Resultados:**

Foi demonstrado: i) é possível ter elevada qualidade e precisão dos dados espaciais utilizados na análise dos resultados de saúde; ii) como as escalas geográficas revelam diferentes graus de detalhe na análise dos resultados de saúde; iii) a escala do “bairro” revelou padrões de mortalidade cardiorrespiratória diferentes da escala habitualmente disponível (freguesia).

**Discussão/Conclusão:**

A nossa análise sugere a relevância em geocodificar os resultados de saúde numa escala mais fina para enfrentar os desafios do sector da saúde e apoiar a tomada de decisões do planeamento, coincidindo estritamente com as necessidades dos cidadãos. Sem correr o risco de perder perspetivas potencialmente importantes, é possível obter uma melhor gestão da saúde, com uma alocação dos recursos otimizada. Assim como é possível uma melhor formulação de políticas, detalhada e esclarecida, permitindo maior equidade de saúde climática na promoção das cidades.

## Introduction

Now that climate emergency is acknowledged [1, 2], the great debate in the scientific community is no longer whether we are dealing with climate change but how we can mitigate and adapt to its effects. It is possible to see climate change as both “the biggest global health threat of the 21st century” [3] and “the greatest global health opportunity” [4]. This double perspective shows that climate change is a complex issue with many consequences and should be viewed beyond an environmental, economic, and technological challenge, as a crucial health issue [5].

Entire populations, rather than only vulnerable subgroups, will be widely affected by climate change [6]. For example, by heatwaves which are expected to become longer, more intense, and more frequent, an increase in mortality and morbidity is expected. In recent years, the remarkable 2003 European heatwave is a reference with its 70,000+ excess deaths [7, 8, 9, 10].

Portugal has a well-documented history of heat-related morbidity and mortality [11, 12, 13]. The 2003 heatwave event caused 1,953 excess deaths, whose largest absolute increase in the number of deaths occurred in the circulatory (758) and respiratory (255) diseases group [14, 15]. The global estimated excess of hospital admissions was 2,576 episodes [13]. The main known vulnerability factors associated with heat are being a child or elderly (over 65 years); having a pre-existing cardiovascular or respiratory disease; medication taking; living alone; socioeconomic deprivation; or living in urban environments [4, 8, 9, 10].

All the concerns about premature and preventable deaths and the issue's relevance are reflected in the global agenda of the Sustainable Development Goals (SDGs) of the United Nations, especially 13.1.1, which measures the number of deaths from natural disasters [16]. It is within this framework of SDG's challenges that we must create a structural change in our societies, aided by health organizations, which must achieve high-quality health systems by acting on the fundamentals of existing systems and investing in improvements to information systems. These improved information systems must (1) contribute to improving politicians' awareness that climate change is a public health imperative, (2) meet the expectations of a more demanding public, and (3) increase trust in healthcare systems [17, 18].

Current advances in computational science enable more effective answers to emerging societal challenges. The improvements in geographic information systems (GISs) have proven to be an efficient tool for mapping the geographical distribution of diseases, its clustering and trends, and spatially modelling environmental, socioeconomic aspects and medical facilities associated with the occurrence of the disease. Currently, GIS has become an increasing trend in various sectors of public health surveillance and spatial epidemiology. It is seen as essential to support spatial decision-making in public health [19, 20, 21, 22, 23, 24, 25, 26]. A noteworthy GIS technique is the geocoding, which has enabled an innovative approach: associating places of residence or neighbourhood scale with health indicators and healthcare needs. Analysis at this scale has become a growing trend in several sectors of health and boosted geomedicine, the personalizing health [19, 27, 28, 29, 30, 31, 32, 33]. Geomedicine shows that health is a continuum and being in good health does not come by accident. It argues that factors in our environment have a considerable impact on our own personal health. Therefore, health relates to the place where we live (work) and where we have lived (worked) in the past. Geomedicine links one's own personal health status to specific geographic factors considering the patient's address and thus gives a robust set of information that helps health professionals to make a better diagnosis. This new approach enables a holistic view of diseases, adding a further dimension to assessing risk and aiding in getting new outcomes, many of which were not expected from the association of environmental data with public health issues/events [27, 34, 35, 36, 37].

Whether individual health behaviour or outcome often results from effects of individuals and neighbourhood, it is crucial to capture accurate results for mapping disease. Accordingly, our health-related behaviour varies depending on geographical contexts, e.g., various levels of urbanization in different areas with distinct natural and built environments, as well as public health policy. This geographical heterogeneity cannot be captured if we map to the scale of the municipalities, not even the parishes. Using health data in predefined administrative units has limited value either for the public who want a holistic overview of a region or for researchers interested in patterns on more detailed geographical scales. The choice of units strongly affects health outcomes [20, 21, 27, 38, 39].

The approach of geomedicine and geocoding allows a re-conceptualizing, going beyond the conventional scales of analysis, offering a more realistic picture of the health/disease of the residents, and thus improving public health information, more equitable healthcare distribution, and identifying urban strategies for promotion of climate health equity in cities, strongly linked to local needs. Already in the climate emergency context, societies should structure themselves to achieve needed public-health objectives through the following process. First, health organisations will provide data on a detailed scale, such as the neighbourhood. Then, computational advances will enable answering the question: where is most urgent to intervene accurately? Based on the answer, we have improved knowledge of health outcomes, and local agencies will optimize economic and human resources to achieve gains in health, economic, social, and urbanistic terms.

As illustration example in this article, we will refer elderly's heat-related cardiorespiratory mortality. This is justified by being a public health problem and mainly because it is well-known that heat-related mortality is not equally distributed among populations and cities/territories [20, 21, 27, 38, 39]. So far, there is no information for Portugal about the elderly's heat-related cardiorespiratory mortality on a neighbourhood scale. Existing studies refer, at most, to the parish scale, not contemplating the differences within them, even when there are parishes of great size. Also, no study was found using geocoding technologies to study such an association. This paper seeks to answer the following questions/deadlocks: “documenting need is not enough; documenting where there is need is critical to intervention strategies” [27]; how does spatial modelling, through geocoding of health outcomes at the neighbourhood scale, provide new insights for healthcare management and urban planning?

The main objective of the article is to raise awareness about the impact of analysing health data at different scales. The results of the analysis are totally different, which can lead to unreasonable decisions in the allocation of resources in healthcare and urban planning. This is exemplified through geocoding. Overall, attention is drawn to modelling with greater equality, which implies additional work, particularly in matters of planning public health services.

The specific objectives of this paper are (i) to deal with the necessary procedures to geocode the Portuguese national e-death certification database. The geocoding process has enough detail to be reproducible and show how to improve the quality and accuracy of health data; (ii) to show the advantageous result of geocoding by mapping the spatial distribution of health outcomes at the neighbourhood scale, i.e., heat-related cardiorespiratory mortality in old individuals; (iii) discuss the most critical challenges that health organisations face in providing detailed data, for confidentiality reasons, as well as discuss the relevance of different scales and its important implications for public health policy and planning.

## Materials and Methods

### Data and Sources

Daily mortality records were provided by the Portuguese Directorate General of Health (Ministry of Health). It was obtained from the National E-Death Certification System in an Excel format. It comprised all causes of mortality between 2014 to part of 2017 (435,291 records), in Portugal, including its autonomous regions (Azores and Madeira). Each mortality record includes the address itself, the house number, the postal code, and the locality. Each record has a six-digit code that corresponds to the district, municipality, and parish. The data of Official Administrative Map of Portugal (Directorate General of the Territory) were used to decode this information.

The Statistics Portugal cartographic base was used to map the data at the municipality, parish, and neighbourhood scale. The information is in vector format at a scale of 1:10,000.

For the geocoding process, the Portuguese spatial buildings' dataset (the Georeferentiation Basis of Buildings [GBBs] from Statistics Portugal) was also used. The information is equally in vector format at a scale of 1:10,000. The latest census data (2011) from Statistics Portugal were used to weight mortality data by the resident population.

### Neighbourhood as a Statistical Unit

In this work, we used the statistical section as congruous with the neighbourhood. A statistical section is a “territorial unit, corresponding to a continuous area of a parish, with about 300 dwellings, intended for housing” [40]. We use the term neighbourhood for simplification purposes.

Note that the parish corresponds to the smaller Portuguese administrative division. Parishes are mandatory subdivisions of municipalities. Each municipality is constituted by a set of parishes [41].

### Geocoding Process

**Fig. 1:**
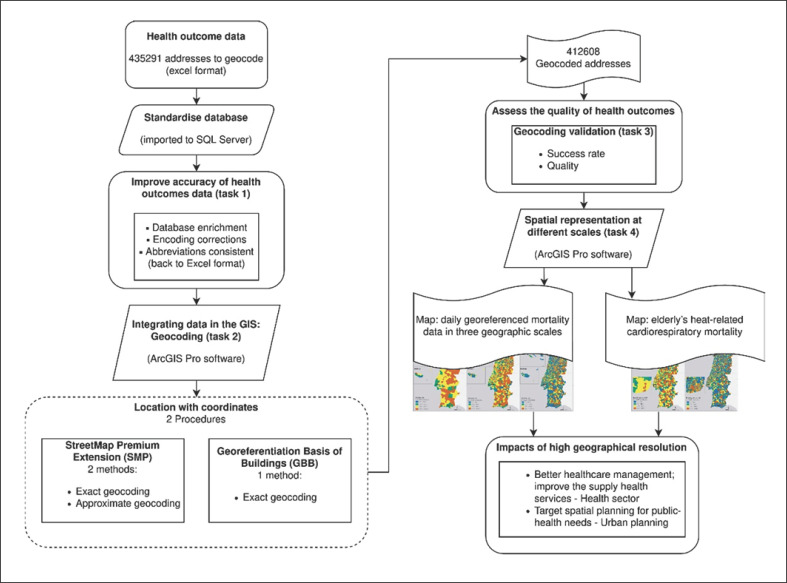
Flowchart of the geocoding process, its validation, and spatial representation of health outcomes.

The geocoding process consisted of thorough data preparation. So, before running the geocoding, there were significant methodological choices and essential steps to avoid potential positional errors. Therefore, we considered various opinions on this subject to ensure good geocoding quality. The following items have a description and reasons for methodological choices for each step: (i) geocoding process quality, (ii) standardisation of the database, (iii) integrating data in the GIS: geocoding, with both procedures used: the StreetMap Premium Extension (SMP) and the GBB, (iv) validation, and (v) spatial representation of health outcomes at different scales. Figure 1 is a flowchart that outlines all the process.

### Geocoding Process Quality

Geocoding process implies converting address data into geographic representations, by matching the address data to geospatial coordinates of reference data, to be spatially displayed within a GIS. Any geocoding process requires reference data, and this step is crucial because the choice of reference data can affect both positional accuracy and match rate. These data must be updated regularly and have maximum completeness and accuracy [31, 42].

The GIS software chosen to perform the geocoding was ArcGIS Pro 2.2.1 using the SMP. The SMP contains commercial street reference data from leading global and local street data suppliers: HERE and INCREMENT P and includes the address locators for most of the world. An address locator can be seen as a library of addresses that indicate the exact location of a searchable address [43, 44]. The address locator specific to Portugal was used.

A sizeable positional error may occur when the criteria are not restricted to obtaining a match, thus increases the matching success rate at the cost of accuracy [45]. The outcome of a geocoding process is a table returning the addresses with a confidence value (a value from 0 to 100) for each potential candidate that has matched the correct location. Some authors consider that matching tolerance of 80 is the level of agreement needed between a searchable address and all the potential candidates [46]. We considered a good match a value greater than or equal to 85. A perfect match is a score of 100 [47].

The validity of epidemiologic research comes from two factors: the match rate of the geocoding (described above and guaranteed) and the positional accuracy of the locations of the geocoded addresses [48]. In the present research, the positional accuracy was defined mainly by choosing the match type for an address that correlated precisely to the searched street (Point Address, Street Address, Street Name, and Postal Ext, each described below).

The quality of geocoding can still be influenced by (i) the quality of the data to be geocoded, i.e., by the completeness of the addresses (the proportion of addresses that can be geocoded, e.g., misspellings or mixed orders of attributes) and (ii) the possible amount of address editing (add attributes to the addresses when the district, country, or parish is missing) which is known as database enrichment and the standardisation (e.g., abbreviations) [32, 49, 50, 51]. This is described in the next section.

### Standardising the Database

The database containing the addresses of deaths was imported into Structured Query Language (SQL) Server. For enriching the database, task 1 in Figure 1, a join was made with the table of the Official Administrative Map of Portugal to obtain the information for each address with a six-digit code (each pair of the three pairs of digits corresponds, respectively, to the district, municipality, and parish).

The next procedure involved standardising the data, including encoding corrections (e.g., Jo?o into João; Bar?es into Barões; Cane?as into Caneças) or making abbreviations consistent (e.g., Ave and Av. into Av). After that, the database was converted back into Excel format and imported into the GIS software.

### Integrating Data in the GIS: Geocoding

The parameterisation of the geocoding process using the SMP, task 2 in Figure 1, is an iterative correspondence between the initial data (addresses of deaths) and the reference data, using the address locator. Note that sometimes, due to mismatches between reference and geocoding data caused by undetected inaccuracies, it can be beneficial to leave some fields out of the process, allowing the address locator to find addresses that had not previously been found, so this procedure was also adopted.

The result of the geocoding process is a layer of points (geocoded addresses) and an output table that contains various fields. The fields most important are score (already spoken, we recall the minimum limit chosen was 85) and Addr_type. The Addr_type is the type of address that was geocoded. Indicating to what kind of feature the address was matched, e.g., we can have a match in the locality or a street. In the latter, the geocoding turns out to be a calculation usually by interpolation along a street segment for which the geographical coordinates of the start and endpoints are known [52]. So, it is possible to study the accuracy of the matched addresses based on the values [43]. The most important values are (i) Point Address (a street address based on points that represent the house and building locations, i.e., address points with associated house numbers and street names. Typically, this is the most spatially accurate match level); (ii) Street Address (an address that represents an interpolated location along a street, given the house number within a range of addresses); (iii) Street Name (like a street address, but without the house number); and (iv) Postal Ext (a postal code with an additional extension, having in total seven digits).

These four values corresponded precisely to the searched address, i.e., the exact geocoding method that established a one-to-one correspondence (they were the most used). The approximate geocoding method means that the address corresponded to the administrative area.

As mentioned, the GBB procedure was also used for geocoding. The GBB is a spatial dataset of point identifiers that have cartographic coordinates and postal codes for each residential building. Through the postal code, it was possible to join the initial data and guarantee the cartographic representation. This procedure is called reverse geocoding, and it was only considered after the SMP because data are updated only every 10 years, in carrying out the census.

All geocoding process was done in four steps (task 2, Fig. 1) and gave priority to the exact geocoding method. The first step was the SMP procedure with the exact geocoding method (all seven filled fields). The second step was with the GBB procedure (exact method). The third was again with the SMP procedure. It involved two steps: the exact geocoding method but with five fields filled (country, postal code, district, municipality, and address) and the approximate geocoding method.

### Geocoding Validation

For measuring the success rate of geocoded data, in task 3 in Figure 1, the ratio of the number of addresses geocoded and the total addresses to be geocoded was used. This calculation was made for three sets of geocoded data: 1) the total number of geocoded addresses by the exact and approximate method, 2) the total number of geocoded addresses by the exact method, 3) the total number of geocoded addresses by the approximate method.

Another calculation was the Sample Representativeness for the three sets of geocoded data, assuming a *p* value = 0.01 [53]. To achieve this threshold, the study needed to include between 15,500 and 16,000 well-geocoded addresses. The present study achieved more than 200,000 matches.

For measuring data quality, the geocoding quality indicator (GQI) was calculated. The GQI has a value associated with each set of geocoded addresses, and that value indicates the proximity of the geocoded location to the correct location of the address. This quality indicator ranges from 0 to −1, with 0 indicating the most accurate proximity and 1 the least [54]. The equation is as follows:

**Figure:**
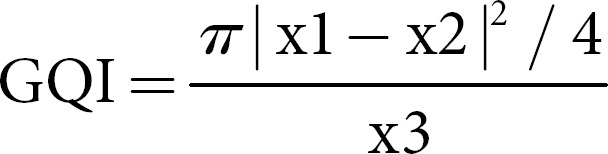

where x1 is the total data volume, x2 is the matching data (exact and approximate geocoding methods 412,608; exact method 208,608; approximate method 204,000), and x3 is the total area of the study site (95,224.7 km^2^).

### Spatial Representation

The result of the geocoding process, the daily georeferenced mortality data, task 4 in Figure 1, provided by the National E-Death Certification System, were represented in Portugal on three geographic scales: the municipality, the parish, and the neighbourhood. For a better spatial comparison, mortality rates were calculated for each administrative area, using the respective census population, defined as the number of deaths per 10,000 inhabitants.

As a case study on the importance of the analysis scale, a specific health outcome is represented, the elderly's heat-related cardiorespiratory mortality, in mainland Portugal and the capital, Lisbon. This city was identified as a good example of the diversity of problems that can occur when making decisions based on public-health data that are too generalised because it is a densely populated municipality. It was considered the daily mortality of the months between May to September, from 2014 to 2017. Maps were constructed based on two of the most vulnerable factors to heat events: (i) individuals aged over 65 years or more and (ii) all diseases of the circulatory (ICD − Cap. IX-I00 to I99) and respiratory system (ICD − Cap. X-J00 to J99) (coded using International Classification of Diseases [ICD] 10th revision). The population census over 65 for each area was used to calculate mortality rates, defined as the number of deaths over 65 per 10,000 inhabitants.

### Additional Data Analysis

As a proof of concept, we used spatial queries to identify the potentially neglected populations, regarded as people living in neighbourhoods with high mortality rates that belong to parishes with medium-low mortality rates.

## Results

### Geocoding

Table 1 shows the geocoding results, in which, of the 435,291 addresses to be geocoded, the SMP procedure was able to geocode 119,540 addresses in a single run. The minimum score selected was 85, and we obtained even the maximum score of 100, showing a high accuracy of geocoded addresses. Then, for the 315,751 records remaining to be geocoded, the GBB procedure was used, obtaining spatial representation for further 37,206 addresses. Between the SMP and GBB, it was possible to geocode 156,746 addresses.

For the remaining 278,545 registers, the SMP procedure was again used, but this time, five fields were filled. It was possible to geocode another 51,862 addresses to the same level of precision − the street − with scores between 83 and 97. In this dataset, the minimum score provided by the software was 83.57. We consider these addresses because they are still above the minimum score of some authors (80) and near the desired 85. It is still a highly satisfactory level of accuracy. In total, 208,608 addresses were geocoded with the highest degree of accuracy.

Then, the SMP was used for the third time, with the approximate method. Another 204,000 addresses were geocoded, increasing the total to 412,608 addresses geocoded out of the total of 435,291.

Only 22,683 unmatched records remained. There were distinct reasons for these unmatched records, including lack of address or postal code, incomplete or even incorrect data, and a lack of correspondence with the SMP and GBB procedures. Although the SMP uses the main suppliers of local and global street data, it does not include all streets, particularly in the country's interior.

Table 2 shows the success rate and quality validations of all geocoding processes. For all addresses geocoded, regardless of the method used, a 94.78% correspondence was obtained from a total of 435,291 addresses (*p* < 0.01). That means the number of geocoded addresses was remarkably high. Besides, Table 2 shows that the GQI was nearly zero, which indicates nearly perfect proximity of the geocoded location to the exact location of the address. That highlights the quality of the geocoding. The values for the exact and approximate methods of geocoding are quite similar because nearly the same numbers of addresses were geocoded using each method. For the exact and approximate methods, the success rate was correspondingly 47.92% and 46.86%, and the GQIs were 0.43 and 0.45, which is also positive.

### Illustration Examples

**Fig. 2:**
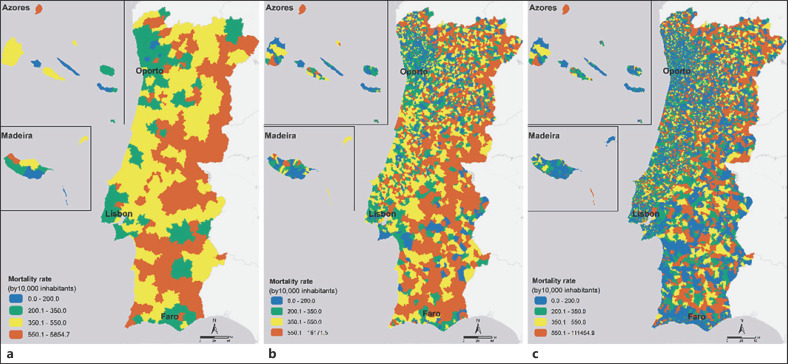
Mortality rates in Portugal between 2014 and 2017 on the scales of the municipality (**a**), parish (**b**), and neighbourhood (**c**).

The three maps in Figure 2 provide an overview of the 4-year mortality rate in Portugal on three scales. Overall patterns from the three scales show a coastal/interior dichotomy of the mortality rate, with the highest values being in the interior. However, the spatial distribution of the mortality rate by neighbourhood scale provides a much higher level of detail. The differences in results between the three scales of geographic analysis are quite remarkable. As we increase the resolution, more details are highlighted in the analysis result. As shown in Figures 2a, b, there are several parishes with very different mortality rates, which were generalised when grouped at the municipality level. This generalisation is even more evident in maps with the scale of the parish and neighbourhood, Figure 2b, c.

**Fig. 3:**
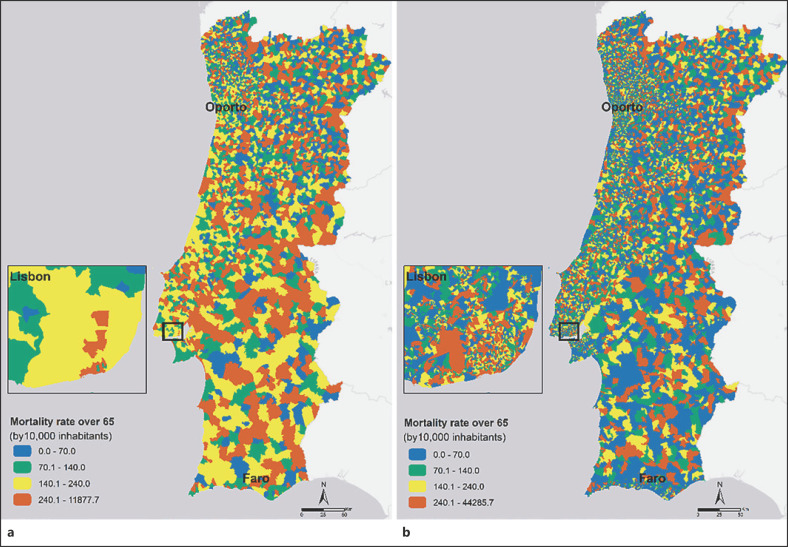
Elderly's (over 65) heat-related cardiorespiratory mortality rates at the parish (**a**) and neighbourhood (**b**) scales from 2014 to 2017.

Similar procedures have been repeated for a more noticeable health outcome to show that geographic resolutions matter in public health analyses. Figure 3 shows the elderly's heat-related cardiorespiratory mortality rate at the parish scale, the only available, and at the neighbourhood scale − both with a zoom to Lisbon.

Once again, it is remarkable that higher resolution analyses have considerably more variability, showing greater geographical detail. Taking Lisbon as an example, the two levels of analysis reveal a very different spatial pattern of cardiorespiratory mortality rate. The most important thing to extract is that there are neighbourhoods that have a low cardiorespiratory mortality rate and are within parishes that have a high rate, highlighting the generalisation problem. Moreover, the most problematic in terms of public health is that there is a high cardiorespiratory mortality rate inside the areas identified with a lower cardiorespiratory mortality rate at the parish level. The essentials are the neighbourhoods with a high cardiorespiratory mortality rate that went unnoticed in a generalist analysis. Therefore, the real dynamics are masked. The identification of these areas at the neighbourhood scale allows acting in a much more efficient way.

If one picks the neighbourhoods (Fig. 3b) with higher mortality rates (>240) and crosses them with the parishes (Fig. 3a) with medium-low mortality rates (<140), we get a total of 484 occurrences. On these neighbourhoods live 305,162 individuals, 48,805 of which are over 65 years old (16%). In these potentially neglected neighbourhoods occurred 2,078 elderly cardiorespiratory deaths during the studied period.

Hence, what is noteworthy is the importance of using several scales of analysis, in an attempt to find the most accurate, the one that best suits the study. The analysis by parishes hides the reality and places barriers to the knowledge of the health outcomes and in the optimisation of resource management and spatial planning.

## Discussion

### Main Findings

According to the World Health Organization (WHO), extreme weather events place a heavy burden on health systems, so those systems need to prepare for the existing challenges and new ones. Health systems were defined as “all the organisations, institutions and resources that are devoted to producing actions principally aimed at improving, maintaining or restoring health” [55]. The vital leadership role of health systems and health administrators in promoting public health amid climate change can also be seen as a moral responsibility and a social obligation. Apart from that, “it is merely a matter of structural transition and policy implementation” [56]. Besides, all the support and influence that the health sector and health professionals can attract from other vital sectors, including government, local political decision-makers, industry, and civil society, is imperative for increasing health information and the creation of mitigation and adaptation measures/policies [55, 56, 57]. This framework motivated this research, which shows the process from upstream, namely, the geocoding of public-health data for an entire country. Then it is illustrated at the different scales by mapping the spatial distribution of health outcomes.

Figures 2 and 3 deal with these challenges. They show the difference between analysing health outcomes with several geographic resolutions. As was shown, a finer scale exposes different spatial patterns, revealing details that are potentially crucial to the knowledge of health outcomes and public health planning. As opposed, analysis at a lower geographical scale may run the risk of hiding potentially significant actions for the population. More important than the overrated areas are the areas that have clearly high mortality rates but go unnoticed in more-general analyses. This situation was exposed when, for Figure 3, data from the neighbourhoods with higher heat-related cardiorespiratory mortality rates in the elderly who are in parishes with medium-low mortality rates were crossed. These neighbourhoods have gone unnoticed in the general analysis and have 48,805 individuals over 65 years. In a climate change scenario with increased occurrence and frequency of heatwaves, this fine scale analysis can potentially identify neglected areas leading to reorganising healthcare management. The operational perspective is different depending on the geographic detail of the phenomenon under analysis. Higher-resolution analyses are preferred because they are closer to reality, to demographic, socioeconomic, urban, and environmental dynamics, and health resources in each area.

Moreover, all this heterogeneity, captured in this study with the neighbourhood scale, influences health. Spatial trends cannot be discovered by using conventional scales of analysis. We argue that using smaller geographic scales can provide more accurate insights about health outcomes. However, it requires the development of health information systems in general.

In any case, the influence of scale on the resulting spatial patterns is known as the modifiable area unit problem. To minimise the effects of modifiable area unit problem, the smallest administrative areas are preferred, once more aggregated data have less variation, smaller variance, and standard deviation [20, 58, 59]. “Aggregation to larger areas should be avoided unless there are good reasons for doing so” [60]. However, in many health institutions, this knowledge does not exist, and therefore there is no discussion about it.

High-scale analysis to map accurate health outcomes implies using suitable methods in GIS to achieve a good quality of the spatial data used in the analysis. Regarding the geocoding process, of the 435,291 records provided (Table 1), 208,608 were geocoded to the street level accuracy, using two procedures: SMP and GBB. The number of unmatched addresses was only 22,683. The remaining 204,000 addresses were also geocoded but not with street-level precision. However, considering that the spatial representation was the neighbourhood, the approximate method of geocoding was enough and did not compromise the reliability and achieves the purpose (even then it was ultimately used).

For the total number of geocoded addresses, the success rate was 94.78%, which is worth noting (Table 2). The GQI value, 0.0043, was also very indicative of quality geocoding because it indicates almost perfect proximity of geocoded location with the actual location of the address.

### Comparison with Previous Studies

Although there was a concern in the methodological choices to reduce the positional error (choice of software associated with the reference data and the minimum match criteria), there is always an associated error. The positional error exists even for the steps where there was a match to the street. Remember that there was a match to the door number (Point Address, Street Address) but also to street segments (Street Name, Postal Ext). However, they are small positioning errors for the address data due to the software interpolation mechanisms and these are errors that depend on the length of the streets (substantial in rural streets) [45]. In any case, aggregation at the neighbourhood level dilutes the error associated with the geocoding process.

However, this dynamic also occurs in other countries. Other authors who have used geocoding mention this: geocoding is generally accurate and has more success in urban areas [42, 45, 46, 49]. Besides, reviews of geocoding spatial errors have noted typical positional errors of 25–614 m [42]. Nonetheless, more significant spatial errors are quite usual in rural areas. It was found that 10% of rural addresses geocoded had errors of more than 1.5 km, and 5% geocoded had errors of more than 2.8 km [61]. The reasons for these most significant errors are several, e.g., (i) rural addresses tend to be less specific, and it is more usual to use unofficial or colloquial place names; (ii) there are larger interpolation errors due to long driveways; (iii) rural route addresses do not exist in commercial street databases with the same frequency than urban routes because there is less population [45, 46].

Concerning the importance of geography in health, much of the recent scientific research goes beyond small-scale administrative boundaries using GIS, involving the neighbourhood's effect on health. Wang even mentions that “just like the importance of personalised medicine revolution in medical care, the ‘individualised neighbourhood effect’ approach will have a lasting impact on public health” [39] (p. 5). Thus, there are several studies of GIS with examples in the field of geomedicine, geocoding, and its potential for human health policies, better healthcare management, and urban planning.

A good example of healthcare management was the study of spatial inequalities in healthcare in low-income urban neighbourhoods. It found that many residents who felt that neighbourhood healthcare services offered poor quality care, suggesting an added perceived distance when trying to access high-quality healthcare services [21]. Likewise, one of the components of the ECHO Project (extension for community healthcare outcomes) reflects a priority in Europe regarding the study of small variations in healthcare [62]. Calovi et al. [20] spatially reorganised the provision of outpatient care services in Italy to provide support to healthcare management and policymakers. It exemplified how GIS can be applied to an integrated structure of administrative healthcare and how valuable they are to improve the efficiency of healthcare service delivery, to the population's needs.

Concerning human health and planning take as an example, a recent study whose objective was to quantify the distance-decay cardiorespiratory diseases risk related to 28 neighbourhood aspects, in a district. The cross-sectional study included home addresses of individuals (all ages) admitted to hospitals, concluding: (i) with a 2,500 m increase in highway length was associated a 46% increase in cardiorespiratory diseases; (ii) 1 km^2^ increase in green areas intra-urban was associated with less two hospital admissions; (iii) those who live ≤500 m from the nearest point of wildfire are more likely to have cardiorespiratory diseases than those who were living >500 m [63]. The effects of the neighbourhood environment on hospital admissions for cardiorespiratory diseases were proved. Similar studies have used geocoding as strategies to reduce the growing cancer burden in Uganda [64], showing the spatially dependent distribution of body mass index [65] or also in environmental exposure assessment, evaluating study participants' residential proximity to environmental exposure sources. The viability of geo­coding residential addresses in epidemiological studies was demonstrated more than 20 years ago [50].

### Study Implications for Healthcare Management and Urban Planning

The importance of the concept of geographical scale, especially in public health, is (has been) well demonstrated. This led us to reflect on the need for a theoretical and methodological improvement in the fields of public health and planning policies. An epistemological and ontological discussion of the concept of scale is lacking, fleeing from merely hierarchical and geometric meanings. We argue that the concept should not only be used but also reflected in this field.

To bring the theoretical-methodological debate of geographical concepts to policies for healthcare and urban planning would be to think of a scalar proposal in cooperation, which would cross the boundaries of the cut, break with territorial boundaries, not only respecting the thresholds of municipalities but the studied phenomenon itself, since its materialisation occurs in one form within the scope of public policy and another in space. The health region, the political-operative space of the health system, is subject to events and should be understood as a space under construction and permanent reconstruction.

Health regions, as planning instruments, appear imbued of a geographical scale, bringing, a priori, the actions of public policies, whether at national, municipal, or parish level and this scale should align with people's needs. The planning decisions should be based on the territorial heterogeneity (which is complex and distinct), maintaining interaction between the different levels of action and thus helping to ensure health equity in the community.

This important cross-border cooperation organisation approaches to the smart governance for health. This concept is about an increased understanding of health, their interdependence, and transitions in the way states and societies work together. Increasingly multidimensional approaches and cooperation will be imperative as health requires an integrated response, whose more informed and knowledgeable policymakers and decision-makers avoid unintended health consequences. After all, diseases and health issues know no geographical boundaries.

So, more than establishing a hierarchy of scales, it is necessary to skip scales through the social, political, and health systems connection that offers a unitary principle for the geographical abstractions that the concept of scale builds. Only from this perspective, spatial planning, policymaking, and the health sector will be efficient in promoting public health.

Our results seem to more accurately answer the question Where? Where is it more urgent to intervene? Future studies should be directed to understanding How? Decision-makers can be now more resourceful and act with more awareness to allocate health resources and increase the sustainability of territories and climate health equity in cities.

Identifying on such a fine scale, the population and vulnerable areas make the scale of the response more appropriate to face the health challenges posed by climate change. Having this sound understanding is a crucial component because climate change remains unabated. The frequency and intensity of heat waves will worsen, as well as the associated cardiorespiratory mortality and hospitalisations, especially in the elderly. This causes a substantial burden on health services and can put them under pressure [66].

One of the key impacts of climate change that influences health outcomes is precisely the exposure to heat, which can lead to disruptions to access to and functioning of health services and facilities, heat stress in hospitalised patients, adverse health outcomes associated with heat stress, and delays in emergency responses. The health sector plays a central role in protecting health from the impacts of climate change. Nonetheless, it should be noted that most health impacts are moderated by the strength of the health system and its ability to manage climate health risks. Therefore, there is a need for overall strengthening of the climate resilience of the health system and preparedness for extreme events in hospitals and healthcare. For human resources, there is a need to develop the technical and clinical capacity of health professionals to meet the changing health needs of their population (different demographic profile and pathologies associated with heat, especially cardiorespiratory diseases) [66, 67]. The type of analysis presented in the manuscript is an advantage and is consistent with the increasingly demanding needs of the near future, becoming essential to intervene in areas of greatest vulnerability faster and better understand the causes of health problems.

However, the pivotal role is not only in the health sector. It requires a response that also works with players outside the health sector to assure coordination and synergies, i.e., under a single climate change strategy and planning. The political engagement, national and local governments, has a central role in public health climate policy, and the most frequent tool has been planning for climate adaptation. The actions of local decision-makers need to be even more targeted to specific health risks, ensure that policies are socially inclusive and progress towards the low carbon transition. A single multisectoral response to deal with the impacts of climate change helps to keep vulnerability to a minimum and increase the resilience of healthcare and urban planning [67, 68].

Regarding the health sector, “the spatial planning of the healthcare system can be defined as a detailed policy to provide healthcare services to all individuals” [23]. Hence, the representation on the detailed scale of the health outcome through geocoding will allow a better understanding of the spatial organisation of healthcare. Linking such information by place can provide new insights into variations in healthcare costs and access, improve the supply of health services, as well as ways to improve the efficiency of healthcare delivery, especially domiciliary care provision, tailored explicitly to citizens' needs. In other words, it is possible to supply more equitable access to resources and overcome spatial disparities by evaluating the current distribution and anticipating future needs [20, 22, 38].

Concerning to urban planning, the detailed scale is also essential, and the results will be useful for city planners. Local policymakers often have limited budgets and knowing in detail the most vulnerable areas in advance is an asset. This combination motivates them to optimise their economic resources, targeting spatial planning for public-health needs, like specific health risks, the resilience of the city, energy efficiency of buildings, and the use of low-carbon construction materials [17, 18, 26, 51].

### Limitations and Future Directions

The preceding is only one example of what health data and the numerous GIS tools can do. It provides new ways to investigate healthcare needs and city planners for small geographic areas. The potential is enormous, and we consider the context of climate emergency and the need of health governance is something that should be considered. However, the use of health data for research purposes is a sensitive issue and therefore manifestly insufficient. There are no doubts about opportunities and gains for public health. The problem is the potential risks, e.g., in terms of access (only the lead investigator or the entire team? In practice, in most cases the data are worked by more than one person) and confidentiality (which in turn raises ethical questions). Most data in health institutions are subject to compliance with the General Data Protection Regulation and confidentiality. Thus, the adversity is that the privacy and confidentiality restrictions limit access to data about health outcomes in most health institutions, especially for individuals or for small areas. Even if confidentiality issues are guaranteed, the majority of institutions are not willing to provide their data so that GIS can create better information for public health and local decision-makers.

Nowadays, the trend is to improve health information systems, provide data always ensuring confidentiality, and associate them with GIS. Thus, the information that is created is extremely useful because it directly contributes to better public health, better information on health outcomes, and more conscious and efficient healthcare management. The same information is also valuable for municipalities and local decision-makers. We take better care of the city and the health of its inhabitants if we also know the city better, in the context of proactive health. Nevertheless, there is a strong legislative framework to overcome. For these data, we obtained consent, but in a similar institution, access to other data that would complement these conclusions was not possible.

This article shows that it is possible to carry out geo­coding accurately and keep the statistical secret (data privacy). After geocoding people's street addresses, the data were generalised to the neighbourhood (but many other techniques exist as well) to ensure statistical confidentiality. Take as examples the mortality locations of Hurricane Katrina that were re-projected to nearby locations [69] or all the different geographic masking strategies for individual-level data, as random direction and fixed radius; random perturbation within a circle; donut masking, among others [70]. This article showed that the path will inevitably be the use of similar data with greater detail if we want to improve public health and improve urban sustainability. However, there are still improvements to be made in legislation/regulation and mainly the dialogue and synergies between researchers in multiple areas and ethics commissions.

Obtaining these data was an asset, exemplifying a much more realistic approach, but there are also other limitations in an analysis on this scale. As already mentioned, larger geographic units tend to mask the internal heterogeneity of the data, but the finer geographical unit, like the neighbourhood, has other issues: the problem of small numbers. The neighbourhoods of aggregation in larger statistical units are a way to avoid the problem of small numbers. Small geographical areas have mostly a smaller population, which generates instabilities and impacts in any analysis. A much more extended analysis period is necessary to confirm the location of the spatial pattern and thus partially solve the problem of small numbers. The use of time series in small areas with a spatiotemporal analysis helps to obtain additional information. However, the work done, using 4 years of data, is a good basis on the effect of spatial data aggregation, namely, in the example of the elderly's heat-related cardiorespiratory mortality rate.

## Conclusions

In this study, we used GIS to geocode deaths in Portugal using the addresses present on e-death certificates, and as a case study, we used the elderly's heat-related cardiorespiratory mortality rate. Analyses using different scales (municipality, parish, and neighbourhood) made clear that the scale of analysis affects the interpretation of health outcomes. Higher scale analyses are more desirable, as more details are uncovered, which are hidden in an analysis with a lower geographical scale. Neighbourhood-scale analysis potentially reveals a more realistic spatial pattern of health outcomes. The importance of the concept of geographical scale, especially in public health, is critical to have information to the higher level of detail than the current one.

This is especially important in the context of climate emergency, which enables health institutions to optimise resources management as well as decision-makers to reduce climate health inequity in the cities. Hence, it is possible to improve health information systems, improve knowledge and accuracy of the spatial pattern of health outcomes, and, in an integrated and multidisciplinary analysis, contribute to better spatial planning and health resource management.

Nevertheless, the biggest challenge is to overcome the strong legislative framework of data confidentiality, even though GIS has several ways to guarantee data protection. The geocoding method used showed that it is possible to improve the quality and accuracy of health data without questioning the current legislation. However, health institutions need to be forward-looking, better organised, and with more people able to deal with technology. In fact, it requires the development of health information systems in general.

## Statement of Ethics

It does not exist.

## Conflict of Interest Statement

The authors have no conflicts of interest to declare.

## Funding Sources

This work was supported by the Fundação para a Ciência e Tecnologia (FCT) and Qart − Soluções de Monitorização e Mapeamento Urbano Ambiental, Lda (grant number PDE/BDE/120452/2016). This publication was supported by ISAMB (Instituto de Saúde Ambiental) (FCT grant UIDB/04295/2020 and UIDP/04295/2020).

## Author Contributions

Liliane Morais participated in the design of the study, cleaned the data, did the process of geocoding, interpreted the results, and wrote the manuscript. António Lopes participated in the revisions and formal analysis. Jorge Rocha participated in the revisions, editing, and geocoding validation. Paulo Nogueira collected the data, and formal analysis, and participated in the revisions and editing.

## Data Availability Statement

The authors cannot provide the data for reasons of confidentiality (these are daily cardiorespiratory mortality records). Hence, the authors have been detailed in the treatment of data, in the methods section, so that it is possible to replicate the study with any other type of data.

## Figures and Tables

**Table 1:** Number of geocoded addresses, according to the applied method and procedure, from the Portuguese national e-death certification database

Total geocoding addresses: 435,291					
procedure	geocoding method	score	geocoded	remaining data	course
SMP	Exact (7 filled fields)	min 85; max 100	119,540	315,751	Step 1
GBB	Exact	Not applicable	37,206	278,545	Step 2
SMP	Exact (5 fields filled)	min 83.57; max 97.84	51,862	226,683	Step 3
	Approximate	min 81.29; max 100	204,000		Step 4
	Unmatched: 22,683 Geocoded addresses: 412,608			

**Table 2:** Validation of geocoded addresses according to success rate and quality

Geocoding method	Total geocoded addresses	Success rate (%)	GQI
Exact + approximate	412,608	94.78	0.0043
Exact	208,608	47.92	0.43
Approximate	204,000	46.86	0.45
